# Motor Functional Reorganization Is Triggered by Tumor Infiltration Into the Primary Motor Area and Repeated Surgery

**DOI:** 10.3389/fnhum.2020.00327

**Published:** 2020-08-14

**Authors:** Riho Nakajima, Masashi Kinoshita, Mitsutoshi Nakada

**Affiliations:** ^1^Department of Occupational Therapy, Faculty of Health Science, Institute of Medical, Pharmaceutical and Health Sciences, Kanazawa University, Kanazawa, Japan; ^2^Department of Neurosurgery, Faculty of Medicine, Institute of Medical, Pharmaceutical and Health Sciences, Kanazawa University, Kanazawa, Japan

**Keywords:** reorganization, neuroplasticity, motor area, awake surgery, glioma

## Abstract

In patients with gliomas, motor deficits are not always observed, even though tumor cells infiltrate into the motor area. Currently, it is recognized that this phenomenon can occur through the neuroplasticity potential. The aim of this study is to investigate the characteristics of motor functional reorganization in gliomas. Out of 100 consecutive patients who underwent awake surgery, 29 patients were assessed as regards their motor function and were retrospectively explored to determine whether positive motor responses were elicited. A total of 73 positive mapping sites from 27 cases were identified, and their spatial anatomical locations and activated region by functional MRI were analyzed. Additionally, the factors promoting neuroplasticity were analyzed through multiple logistic regression analysis. As a result, a total of 60 points (21 cases) were found in place, while 13 points (17.8%) were found to be shifted from anatomical localization. Reorganizations were classified into three categories: Type 1 (move to ipsilateral different gyrus) was detected at nine points (four cases), and they moved into the postcentral gyrus. Type 2 (move within the ipsilateral precentral gyrus) was detected at four points (two cases). Unknown type (two cases) was categorized as those whose motor functional cortex was moved to other regions, although we could not find the compensated motor area. Two factors for the onset of reorganization were identified: tumor cells infiltrate into the primary motor area and repeated surgery (*p* < 0.0001 and *p* = 0.0070, respectively). Our study demonstrated that compensation can occur mainly in two ways, and it promoted repeated surgery and infiltration of tumor into the primary motor area.

## Introduction

It is known that paresis following brain damage can be partly recovered (Sawner and LaVigne, 1970). Recent progress in neuro-imaging and intraoperative monitoring has revealed that neural networks can change dynamically (Schulz et al., 2016; Lefebvre et al., 2017). The mechanism that rearranges cerebral networks after brain damage has been termed as neuroplasticity or reorganization (Duffau, 2006).

Several reorganization patterns exist. In low-grade gliomas, the patients can occasionally maintain normal functioning, even after tumor cells have infiltrated into an anatomically functional area (Desmurget et al., 2007; Hayashi et al., 2014; Bourdillon et al., 2017). Awake surgery is one of the surgical methods with intending maximum safe resection to resect brain lesion including eloquent area, such as movement or language. Glioma surgery under awake condition monitoring provides a unique validation opportunity with direct evidence. Actually, we occasionally encounter the fact that functional area may move after surgical invasion. For instance, the motor area identified in the initial surgery can be resected after a few months or years, because the positive mapping sites of movement disappear in the second surgery (Duffau, 2006; Southwell et al., 2016; Ghinda and Duffau, 2017). In rare cases, motor areas that were not found in the initial surgery suddenly appear during subsequent surgeries (Duffau et al., 2000; Duffau, 2001). This is considered as intraoperative unmasking, that is, rapid compensation. In some cases, after amputation, the sensorimotor area of a lost body part can replace that of another intact body part (Kuehn et al., 2017). Moreover, with regard to motor function, it is possible that the contralateral motor area can compensate (LeRoux et al., 1991; Ius et al., 2011; Vassal et al., 2017).

Unfortunately, the reorganization of the motor area does not always occur, and sometimes patients suffer from paresis as a result of damage to the motor area. A recent study by Herbet et al. (2016) investigated the neuro-plastic potential of the compensation mechanism and found that the plasticity of the cortical level is more probable than that of the subcortical area, except for the primary area including the sensory, motor, and auditory areas. Additionally, a slow-growing lesion and surgical invasion are considered as functional shift triggers from clinician’s experience (Desmurget et al., 2007; Bourdillon et al., 2017; Ghinda and Duffau, 2017). All things considered, it is unclear under which condition the compensation mechanism will occur and with which pattern of reorganization.

This cross-sectional study investigated the motor functional reorganization characteristics of gliomas using intraoperative findings and functional magnetic resonance imaging (fMRI). In the current study, we defined motor functional shift as when motor area moved outside of the original anatomical location, as previously described (Duffau et al., 2000, 2006). We found two types of reorganization and two conditions that promote the compensation mechanism in the primary motor area with statistical evidence. The findings of this study are expected to be useful for researchers engaging in neuroscience, as well as neurosurgery, neurology, and rehabilitation.

## Materials and Methods

### Patients

**FIGURE 1:**
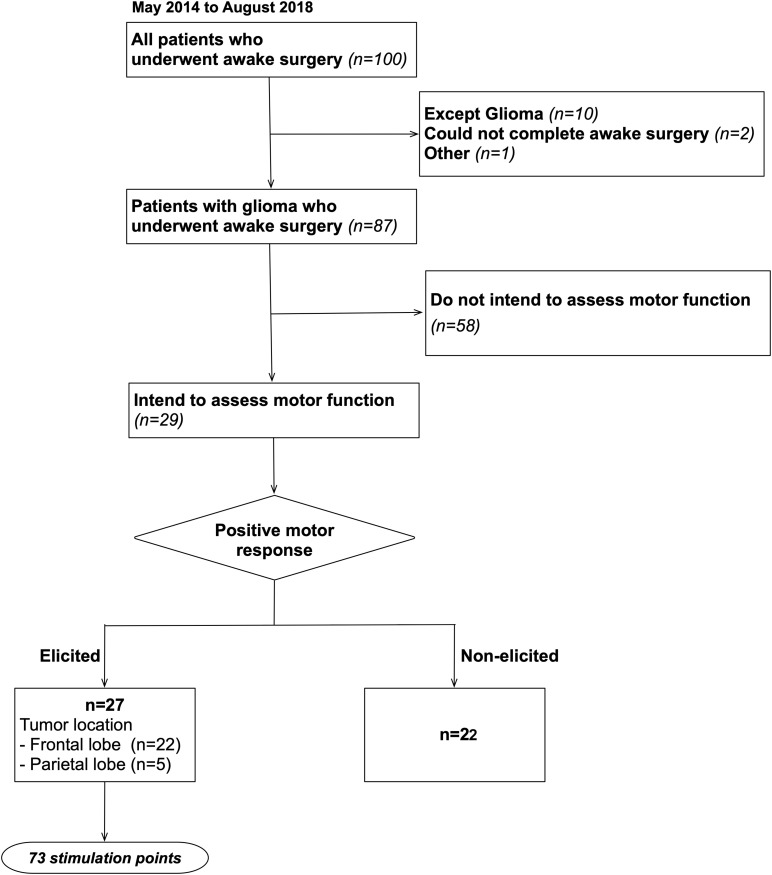
Inclusion criteria. A total of 100 patients who underwent awake surgery in our university were included, and 87 of these patients matched our inclusion criteria. Then, we studied 87 patients retrospectively and found that 29 patients were operated in awake condition intending to identify motor area.

This study recruited a total of 100 patients who underwent awake surgery for the resection of brain tumor in our hospital between May 2014 and August 2018. From these 100 cases, 87 cases who completed surgery under awake condition for glioma matched our inclusion criteria. Of these, 29 cases were intended to assess motor function during awake surgery (details are shown in Figure 1). To note that, our patient group was not limited to patients whose tumors infiltrate into the primary motor area. Written informed consent was obtained from the individual(s) or next of kin for the publication of any potentially identifiable images or data included in this article. Additionally, this study was conducted in accordance with the guidelines of the Internal Review Board and Declaration of Helsinki and received the approval of the medical ethics committee of our university (Approval number: 2994).

### Awake Surgery

All patients were operated using the “asleep–awake–asleep” technique, and the cortical and subcortical brain mapping was accomplished through direct electrical stimulation (DES) (Duffau et al., 2002). A bipolar electrode with 5-mm-spaced tips delivering a biphasic current (pulse frequency of 60 Hz, single-pulse phase duration of 1 ms, amplitude between 1.5 and 6 mA) was used in all cases. To identify the central sulcus, we used the somatosensory evoked potential (SEP) or DES to the sensory and motor area under the awake condition. Awake surgery aiming to preserve language and sensorimotor functions is now considered as the gold standard, and it is covered by national insurance in Japan. Nowadays, neuropsychological function such as visuospatial cognition, emotional function, and working memory are also known to be preserved during awake surgery (Thiebaut de Schotten et al., 2005; Kinoshita et al., 2016; Yordanova et al., 2017; Nakajima et al., 2018). However, intraoperative assessment for neuropsychological function is still challenging in glioma surgery and long-term functional and oncological outcome is not fully understood. In this study, several tasks including motor and superficial perception were performed intraoperatively and were carefully chosen in each patient. Importantly, to achieve appropriate onco-functional balance for all glioma patients, we first resected the central part of the tumor, that is, the enhanced lesion, and then performed intraoperative assessments on the extended resection (Esquenazi et al., 2017).

With regard to intraoperative motor functional assessment, the patients were asked to simultaneously move their fingers and elbow. During DES, several motor symptoms, such as dystonic movement, motor arrest, acceleration, and coordination disorder, were elicited. Among them, the symptom of dystonic movement is represented by a pyramidal tract sign, and it is determined as positive motor mapping (Rech et al., 2016). We adopted continuous movement of upper limb so as to distinguish positive and negative motor symptoms. Negative motor response, which is elicited by stimulation to negative motor area, is defined as cessation of voluntary tonic muscle contraction or of rapid movements without loss of consciousness. It can be clearly distinguished from pyramidal tract sign (Schucht et al., 2013; Kinoshita et al., 2015). The area where negative motor responses are elicited represents negative motor area, such as the supplementary motor area. Its damage may cause temporal motor deficit, but not permanent deficit (Nakajima et al., 2019).

All points were stimulated a total of three times, and abnormal responses were observed in two or all three sets, which were evaluated as positive intraoperative responses. Patients were operated by the experienced neurosurgeons (MK and/or MN).

### Functional Assessment

The motor function of all patients was assessed using the Brunnstrom recovery stage (BRS) (Sawner and LaVigne, 1970), which is widely used to assess the recovery stage of paresis. The BRS consists of six levels ranging from Stage 1, which indicates the most severe and flaccid paresis, to Stage 6, which indicates very mild, but not normal, paresis. Motor assessment was performed 1 week and 3 months pre- and post-operation.

### Magnetic Resonance Imaging and Postprocessing

Structural magnetic resonance (MR) imaging was carried out for all patients as a part of standard care at the pre- and up to 3-month post-operative periods. The MR images were obtained using conventional high-resolution three-dimensional (3D) T1-weighted sequences on a 3.0-Tesla MR imaging scanner (Signa^TM^ Excite HDx 3.0T; GE Healthcare, Little Chalfont, United Kingdom). Each MR image was conformed to the MNI template using SPM12^1^, which is implemented in MATLAB^2^. Each individual MR image transformed into MNI space was checked to determine accuracy of transformation with comparing anatomical landmark, such as sulci and brainstem. Then, the resection cavities were reconstructed manually using MRIcron software^3^. Each reconstruction was first constructed by the first author (RN) and then systematically checked by a neurosurgeon (MK). The resection cavities were overlaid onto the template of the Brodmann area (BA), and the overlapped voxels were automatically calculated. Through these procedures, we determined whether BA4/6 were resected in each patient. Additionally, the degree of infiltration for each patient was classified as diffuse, localized, or minimal using the diffusion tensor imaging data, according to a previous study (Mohsen et al., 2013).

### Spatial Topography of Stimulations

In awake surgery, 73 positive mappings of motor points were obtained with DES during the movement task and subsequently investigated (Figure 1). Step 1: The positive mapping sites were plotted on the corresponding original 3DT1 images in each patient using operative reports and intraoperative video records using iPlan Stereotaxy 3.0 software (BrainLab). The exact locations of the DES points were determined in accordance with their spatial relationships to various anatomical landmarks (gyri, sulci, vessel, midline, and/or lateral ventricles) (Tate et al., 2014). The plotted positive mapping sites on 3D brain can be also automatically identified on original 2D-MR images. Step 2: Each positive mapping site on original MR images was transferred to normalized T1 images. Step 3: The spatial location of the positive motor mapping sites was overlaid on the 3D MNI template using the MRIcroGL software^4^. We checked again the anatomical location of positive mapping sites on the 3D MNI template by comparing original operative records. Schematic views of these steps are summarized in Supplementary Figure S1. Each reconstruction was first carried out by the RN, and then systematically checked by a neurosurgeon (MK).

### Functional MRI

Preoperative fMRI was performed routinely for all patients. We employed finger tapping (index finger on the thumb tip using contralateral side of brain tumor) and tongue movement (tongue retraction movement) as a motor functional task (Grabski et al., 2012; Shirani et al., 2017). Additionally, picture naming was carried out to identify the region related to language and speech. We asked all participants to practice before session and monitored inside the MRI theater whether they perform the task effectively. The fMRI was performed with single-shot gradient-echo echo-planar imaging, as follows: block design; repetition time of 3000 ms; echo time of 35 ms; flip angle of 90°; field of view of 200 mm × 200 mm; slice thickness/gap of 3 mm/0 mm; acquisition matrix of 64 × 64; total acquisition time of 3 min and 15 s (five dummy scans). We used BrainWave (GE Healthcare^5^) to analyze and create an activation map with generalized linear model (uncorrected).

### Statistical Analysis

To analyze the factors influencing the motor functional shift, first, we carried out univariate analysis using the Wilcoxon test or Pearson’s chi-squared test with consideration to the Bonferroni correction. Additionally, logistic multiple regression analysis was performed. In this analysis, all variables associated with the independent values were selected using stepwise methods (*p* < 0.15). The probabilistic explanatory variables were selected based on previous studies as follows: age, WHO grade, histology, status of IDH-1 mutation, degree of infiltration, surgical history, tumor location in BA4, tumor location in BA6 but not BA4, and preoperative paresis (Duffau, 2006; Gutchess, 2014; Southwell et al., 2016; Cirillo et al., 2019). Objective value was whether motor functional shift occurs regardless of how to move. The non-parametric test was used in the current study since our data did not follow a normal distribution. All statistical analyses were performed using statistical analysis software (JMP, version 14.3.0; SAS Institute, Inc.).

## Results

### Motor Functional Outcome

Before surgery, paresis was not observed in any patient. Just after surgery, moderate and slight paresis was observed in one (Case 5) and five cases (Cases 2, 4, 6, 7, and 8), respectively (Supplementary Table S1). Paresis just after surgery was inevitable in some cases, even though motor function was intended to preserve during awake surgery, since invasion of surgery came next to motor area. In that case, motor function was expected to recover during 3 months postoperatively, because critical region for motor function was preserved (De Witt Hamer et al., 2012). As expected, at 3 months after the surgery, three patients (Cases 2, 5, and 8) had very slight paresis and five patients had quite normal motor function. All patients were independently capable of living their daily lives.

### Intraoperative Positive Mapping Sites

**FIGURE 2:**
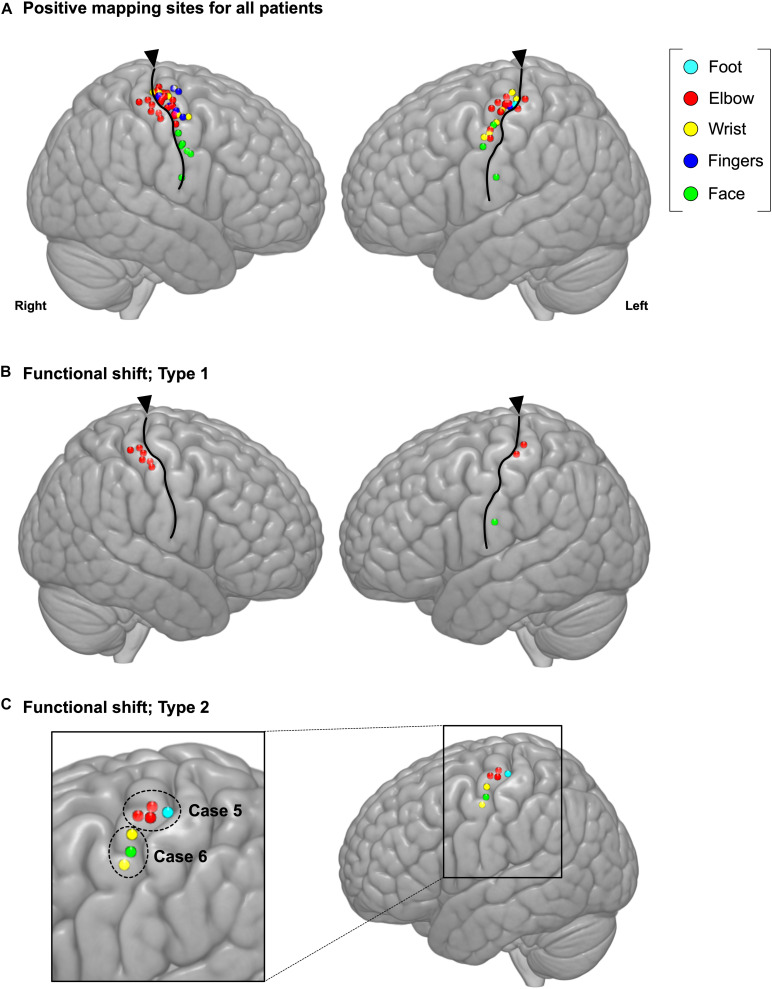
Positive mapping of motor function sites during awake surgery. **(A)** The cerebral regions were directly stimulated during awake surgery to identify the motor area. When involuntary movement or dystonic movement was observed (positive mapping), these regions were defined as the motor area. We marked the location of positive mapping sites and body parts for which the symptoms were elicited. Upon analyzing the characteristics of the motor area’s functional shift, we identified two functional shift types: **(B)** Type 1 (move to ipsilateral different gyrus) and **(C)** Type 2 (move within the ipsilateral precentral gyrus). Cyan: foot; red: elbow; yellow: wrist; blue: fingers; green: face; triangles: location of central sulcus.

The spatial topographies of the intraoperative positive motor site mappings are shown in Figure 2A. A total of 73 motor points from 27 cases were found. Patient details are shown in Table 1. Among all motor positive mapping points, 60 points (21 cases) were found in place, while 13 points were found to be shifted from anatomical localization. When we analyzed the localization of the motor area, we found that the motor functional reorganization can be divided into three types (Table 2): Type 1 (move to the ipsilateral different gyrus, Figure 2B), Type 2 (move within the ipsilateral precentral gyrus, Figure 2C), and Unknown type (there are evidence to move, but the destination cannot be found). The MNI coordinates for all positive mapping sites are listed in Supplementary Table S2.

**TABLE 1:** Demographic and clinical characteristics of participants who were intended to assess motor function.

**Factors**	**Number of patients**
Age	53.3 ± 12.7 (29–72)
Sex; male/female	21/8
WHO grade	II 6, III 12, IV 11
Histology	
Diffuse astrocytoma	4
Oligodendroglioma	2
Anaplastic astrocytoma	4
Anaplastic oligodendroglioma	8
Glioblastoma	11
IDH-1 mutation; wild type/mutant	10/19
Tumor location; frontal/parietal	24/5
Surgical history; initial/2nd or subsequent surgery	17/12

*IDH, isocitrate dehydrogenase.*

**TABLE 2:** Patients’ characteristics whose motor function shifted.

			**Diagnosis**				**Location**	
**Case**	**Ages**	**Time of surgery**	**(IDH-1 mutation)**	**Infiltration pattern**	**Tumor location**	**Pre-op paresis**	**of motor area**	**Shift type**
1	30s	2nd	OD (mutant)	Minimal	BA4	Normal	Pre-CG, post-CG	Type 1
2	30s	Initial	GBM (wild type)	Localized	BA4	Normal	Post-CG	Type 1
3	40s	Initial	AA (mutant)	Minimal	BA4	Normal	Post-CG	Type 1
4	50s	2nd	AA (mutant)	Minimal	BA6 but not BA4	Normal	Pre-CG, post-CG	Type 1
5	60s	>2nd	GBM (mutant)	Localized	BA4	Normal	Pre-CG	Type 2
6	70s	2nd	GBM (wild type)	Diffuse	BA6 but not BA4	Normal	Pre-CG	Type 2
7	60s	>2nd	AO (mutant)	Localized	BA6 but not BA4	Normal	Unknown*	Unknown
8	60s	>2nd	AA (mutant)	Localized	BA6 but not BA4	Normal	Unknown*	Unknown

M, male; F, female; BA, Brodmann area; OD, oligodendroglioma; AA, anaplastic astrocytoma; GBM, glioblastoma; pre-CG, precentral gyrus; post-CG, postcentral gyrus; IDH, isocitrate dehydrogenase. *Indicates that positive mapping sites are moved in repeated surgery. In the initial surgery, positive mapping sites of motor areas were observed in precentral gyrus, but they were not found in the second surgery.

### Three Categories of Reorganization

#### Type 1: Move to the Ipsilateral Different Gyrus (Figure 3)

The Type 1 functional shift of motor functioning was found at nine points from four cases, which were located in the postcentral gyrus. Positive motor responses were observed in all these four cases’ sensory area, whereas we could not find positive motor responses in sensory area except for these cases. In Cases 1 and 2, the activated area was found in the postcentral gyrus, rather than in the precentral gyrus, using preoperative fMRI. In Case 3, both the precentral and postcentral gyri were activated in the movement task. However, in Case 4, the activated area was not observed in the postcentral gyrus, but was instead found within the resection cavity of a previous surgery from 15 years ago. Though BOLD signals were not obtained within the cortical area from fMRI in Case 4, we prioritized the result of DES in the current study, and we classified the case as type 1. Before the cortical mapping, the central sulcus was determined using the SEP in Case1, and the intraoperative DES in Cases 2 to 4.

**FIGURE 3:**
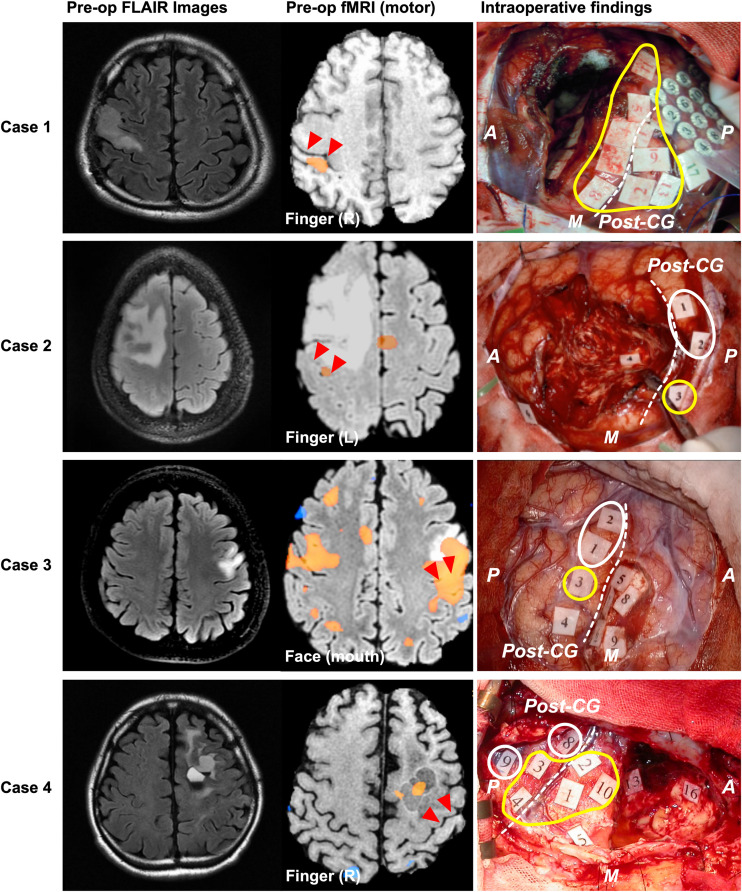
Patients whose motor functions shifted in Type 1. The left, middle, and right columns show the preoperative FLAIR images, preoperative functional MRI for movement, and intraoperative findings, respectively. In Cases 1 and 2, blood oxygenation level dependency (BOLD) was observed in the postcentral gyrus during the movement task. However, in Case 4, BOLD dependency was observed within the resection cavity of previous surgery, but not in the precentral or postcentral gyrus. Positive mapping sites for movement were found in the postcentral gyrus of four patients during awake surgery. In Cases 1 and 4, positive motor responses were elicited in both the precentral and the postcentral gyri. However, in Cases 2 and 3, positive motor responses were only found in the postcentral gyrus. The red triangles in the middle column and white broken line in the right column indicate the central sulcus; the yellow circles indicate positive mappings for motor function sites; the white circles indicate positive mappings for sensory sites. A: anterior; P: posterior; M: medial side; CG: central gyrus. Here, we show Tags and symptoms elicited by electrical stimulation except for sensory and motor. Case 1: Tag 17, sensory; Case 2: Tag 4, negative motor response (foot); Tag 6, emotion recognition; Case 3: Tags 4, 6, and 7, dysarthria; Tags 5 and 9, anarthria; Tag 8, negative motor response; Case 4: Tags 5, 13, and 16, paraphasia.

#### Type 2: Move Within the Ipsilateral Precentral Gyrus (Figure 4)

A total of four points from two cases were defined as the Type 2 functional shift. The motor area was obviously found at a different anatomical location compared with the original functional localization of the primary motor area. In this step, we first found the precentral knob in the axial slice of the MR images, and then determined the position as the motor area of the hand (Yousry et al., 1997). In Case 5, the motor area of the elbow and shoulder was elicited in the foot area. Accordingly, in Case 6, the motor area of the face was identified between the hand area and wrist area, that is, the original hand area (Supplementary Video S1).

**FIGURE 4:**
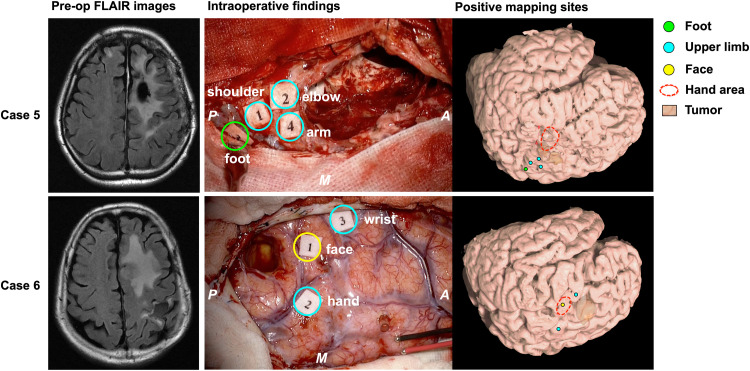
Patients whose motor function moved according to Type 2. The left columns show the preoperative FLAIR images; the middle columns show the intraoperative findings; the right columns show the positive mapping sites for each patient’s brain reconstructed from structural MRI. In Case 5 (upper column), the motor area of the upper limb was observed next to the foot area. In case 6 (lower column), the face area was found between the hand and the wrist area. Green circle: positive mapping site of foot; cyan circles: positive mapping sites of upper limb including shoulder, arm, elbow, hand, and wrist; yellow circle: positive mapping site of face; red broken line: knob area in the precentral gyrus, namely, the hand area. A: anterior; P: posterior; M: medial side.

#### Unknown Type (Figure 5)

**FIGURE 5:**
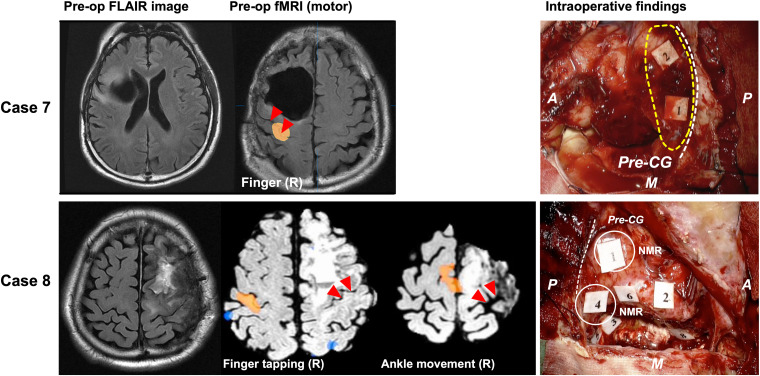
Patients whose motor functioning shifted, but the destination of the shift is unknown. The left column shows the preoperative FLAIR image. In case 8 (lower column), the functional MRI investigation for the movement task of the right finger and the foot revealed the BOLD in the precentral and postcentral gyri of the right cerebral hemisphere (middle two columns). During surgery, involuntary movement was not observed in the motor area, but negative motor response was observed (Right lower column, white circle). The red triangles in the middle column and white broken line in the right column indicate the central sulcus; The yellow broken line in the upper right column indicates motor area recognized as positive motor response in previous surgery; A: anterior; P: posterior; M: medial side; broken white line: central sulcus. Tags indicate intraoperative findings as follows: Case 7: Tags 1 and 2, anarthria; Case 8: Tags 1 and 4, negative motor response (NMR) in cortical level; Tag 2, paraphasia; Tags 3 and 5, NMR in subcortical level; Tags 6 and 8, paraphasia; A: anterior; P: posterior; M: medial side.

In two cases, existing evidence demonstrated that the motor area moved from the proper position, although the motor functional area was not found in the operative field. In Case 7, we confirmed that the motor area was located in the primary motor area via DES during a previous surgery (Supplementary Figure S2). However, we did not observe positive response in the next surgery at the identical region where involuntary movement had been elicited (Figure 5, upper columns). The patient’s motor function was quite intact after surgery. We speculated that the motor area shifted to another gyrus, because the postcentral gyrus was active in the fMRI.

In Case 8, the preoperative fMRI revealed that the right (not left) precentral to postcentral gyri were activated during the movement of the right upper and lower extremities. Contrary to our expectation during awake surgery, positive response for movement of right upper and lower extremities was not observed in the operative field; instead, negative motor response was observed (Supplementary Video S2). Note that positive motor response by the DES on the contralateral hemisphere could not be found because it was outside the intraoperative mapping area.

### Factors Related to Motor Functional Shift

We carried out the Wilcoxon test and Pearson’s chi-squared test with consideration to the Bonferroni correction. As a result, one factor, namely, tumor location (involving BA4 as a lesion), was identified with statistical significance (*p* = 0.0019, Table 3). Next, we conducted multiple regression analysis using stepwise method, and four factors including age, degree of infiltration, surgical history, and tumor location were detected. Finally, two factors were found to be statistically significant in promoting reorganization: the inclusion of BA4 as a lesion and repeated surgery (*p* < 0.0001 and *p* = 0.0070, respectively). Importantly, age and malignancy grade were not influenced by the occurrence of functional reorganization.

**TABLE 3:** Related factors of motor functional shift in patients with frontal gliomas.

	***p*-Value**	
		**Multiple logistic**
	**Univariate**	**regression analysis**
	**analysis**	**with stepwise method**
Age	0.93	0.15
WHO grade	0.47	–
Histology	0.10	–
IDH-1 mutation	0.16	–
Degree of infiltration	0.67	0.57
Surgical history; initial/2nd or subsequent surgery	0.083	0.0070**
Involving primary motor area (BA4)	**0.0019^∗∗^**	<0.0001****
Involving pre-motor area (BA6), but not BA4	0.14	–
Preoperative paresis	0.72	–

Bold, significant considering Bonferroni correction; **p < 0.01; ****p < 0.0001. For univariate analysis, Wilcoxon test or Pearson’s chi-squared test was used. Multiple regression analysis: *R*^2^ = 0.67 (*p* = 0.0004). BA, Brodmann area; IDH, isocitrate dehydrogenase.

To support these results, functional shifts were found in 16.7% of the patients after the initial surgery and in 41.7% of the patients after the second or subsequent surgeries (Supplementary Figure S3A). Additionally, motor functional reorganization occurred in 100% of patients whose lesions infiltrated into the primary motor area (BA4) and in 28.6% of patients whose lesions extended adjacent to a motor area such as the pre-motor area (BA6). Importantly, when the tumor was located in areas other than the BA4 or BA6, motor functional shift did not occur (Supplementary Figure S3B).

## Discussion

This study investigated the motor functional reorganization characteristics of gliomas according to the intraoperative findings obtained during awake surgery and fMRI. Thus, three categories of motor functional shift were identified: (1) move to ipsilateral different gyrus, (2) move within the ipsilateral precentral gyrus, and (3) unknown type (there are evidence to move, but the destination cannot be found). Using multiple logistic regression analysis, two factors promoting the neuroplasticity of the motor area were determined: the infiltration of tumor cells into the primary motor area and repeated surgery. Our study that is simple and uses a direct and novel approach classified the reorganization pattern for the primary motor cortex and revealed the factors promoting the neuroplasticity of motor areas with statistical evidence.

### Categories of Motor Functional Shift

#### Type 1: Move to Ipsilateral Different Gyrus

Various studies have reported that the motor area can move to an ipsilateral different gyrus (Fandino et al., 1999; Duffau et al., 2000, 2006, Duffau, 2001). However, it is not commonly known that the motor area can also move to the postcentral gyrus beyond the central sulcus. In support of our results, three previous single-case studies have reported that the motor area moves to the postcentral gyrus when triggered by brain damage or surgical invasion. Two of these cases used fMRI (Jang et al., 2002; Hayashi et al., 2014), and another case used intraoperative DES (Duffau et al., 2000). The popular hypothesis for the mechanism is the superficial short fiber or U-fiber that connects the adjacent gyri (Maeda et al., 2013; Guevara et al., 2017). In fact, approximately all people have the U-fiber, which communicates with the primary motor and sensory cortices. Typically, however, motor symptoms are not elicited in the sensory area, as revealed by our results. Thus, we consider that motor symptoms can be elicited in the postcentral gyrus when the motor area is infiltrated by tumor cells. In this case, the sensorimotor connectivity will be strengthened owing to the pre-operative unmasking or contribution of the accessory pathway (Duffau, 2009).

The cerebral cortex, which includes the primary motor and sensory areas, has a six-layered structure and the cellular structures of the adjacent gyri are obviously different (Kahle et al., 1981). The primary motor area consists of the expanded pyramidal cell and the degenerate granular cell, while the primary sensory area consists of the expanded granular cell and the degenerate pyramidal cell. Moreover, it is previously known that motor and sensory areas are not always strictly divided by central sulcus because both gyri have connection to each other, and unexpected results are occasionally found especially in the super-sylvian region (Horsley, 1909; Van Buren, 1983; Darian-Smith et al., 1993). Hence, physiologists have indicated that a motor response may be elicited when the postcentral gyrus is strongly stimulated (Kahle et al., 1981). This study applied stimulation between 1.5 and 6.0 mA, which is the minimum stimulation intensity required to elicit motor symptoms. Moreover, among 87 cases, the motor symptoms in the postcentral gyrus were evoked only in four cases, wherein the tumor was located in BA4 or adjacent to BA4, as shown in Figure 2. Considering other evidence from fMRI, the probability of symptom provocation derived from just the cellular structure may be quite low.

#### Type 2: Move Within the Ipsilateral Precentral Gyrus

In this study, the functional localization shifted within the primary motor area at two points. In the 1930s, Penfield et al. (Penfield and Boldrey, 1937) found that there was functional localization within the motor area. More recently, somatotopy through DES has been reported (Maldonado et al., 2011; Rolland et al., 2018), and it mostly corresponds to the findings reported by Penfield (Penfield and Boldrey, 1937). It is well known that somatotopy can be changed depending on the frequency of use, which is termed as use-dependent plasticity (Butefisch et al., 2000; Oouchida et al., 2016). The brain mapping mechanism is re-drawn according to repeated usage, such as in motor training or after amputation (Papale and Hooks, 2018; Bramati et al., 2019; Gunduz et al., 2020). Duffau (2001, 2006) have reported a rare case wherein the motor area moved within the precentral gyrus after surgical invasion, which is known as acute unmasking. Generally, this type of brain reorganization has been thought to occur according to afferent input. This study revealed that somatotopy can shift when changes occur in the brain.

As per the previous section, the hypothesis that motor functional shift occurs via U-fiber is now considered as the most prepotent mechanism of reorganization (Maeda et al., 2013; Guevara et al., 2017). Though U-fiber has been considered as the short fiber that connects adjacent gyri, very recently we firstly found U-fibers connecting intragyral points (Shinohara et al., 2020). Our finding might indicate from structural aspects that functional localization can move within gyrus.

#### Unknown Type

In two cases classified as unknown type, there are evidences that motor area shifted from the original anatomical primary motor area to others. Nonetheless, some of the real motor area could not be found since motor area might shift outside of the operative field. There is a possibility that the contralateral hemisphere may compensate when the unilateral sensorimotor area is damaged (LeRoux et al., 1991; Bulubas et al., 2020). Nowadays, the compensation of the contralateral hemisphere is considered to act through trans-callosal pathways (Ius et al., 2011). In our patient group, there existed only one patient with high-grade glioma whose motor area shifted to outside the operative field. Possibly, the dynamic change of the neural network may be a reorganization strategy for the rapid growth of high-grade glioma (Fandino et al., 1999; Majos et al., 2017).

### Factors Promoting Motor Functional Shift

Tumor location: This study statistically significantly found that the infiltration of tumor cells into BA4 was one of the factors contributing to the occurrence of functional reorganization. In fact, when the tumor extended to BA4, different types of functional shift occurred in 100% of our patient group. According to previous research wherein brain plasticity occurred in the cortical level, lesions were found in the primary motor area for all subjects including humans and rats, without exceptions (Donoghue et al., 1990; LeRoux et al., 1991; Fandino et al., 1999; Duffau, 2001; Jang et al., 2002; Majos et al., 2017). Hence, it can be said that the infiltration of tumor cells into BA4 is an important factor contributing to the motor functional shift.

Surgical history: In line with previous findings (Duffau, 2006; Southwell et al., 2016; Ghinda and Duffau, 2017), the statistical results obtained by this study revealed that the motor area may shift in the second surgery, or in subsequent surgeries, rather than in the initial surgery. The phenomenon whereby the area that was positively mapped in the initial surgery is missing or moving in the second surgery is considered to occur as a result of surgical invasion (Hayashi et al., 2014; Southwell et al., 2016; Ille et al., 2019; Bulubas et al., 2020). The mechanism of neuroplasticity enables the neurosurgeon to resect the motor area while preserving motor function. However, since the actual functional and structural mechanism is not fully understood, further study will be required to reveal the phenomenon that was seen in clinical practice.

It is known that reorganization can occur in slow-growing glioma (Duffau, 2006; Desmurget et al., 2007); however, various studies have revealed that the reorganization mechanism may also develop in glioblastoma (Fandino et al., 1999; Southwell et al., 2016; Majos et al., 2017). In this study, the malignancy grade and infiltration pattern did not influence the emergence of reorganization. Interestingly, in glioblastoma patients, the contralateral motor and supplementary motor areas are known to activate as a result of compensation after damage to the motor area (Fandino et al., 1999; Majos et al., 2017). These dynamic changes of neural networks may enable a rapidly progressing high-grade glioma to shift the functional area (Duffau, 2009).

### Limitation

This retrospective study investigated the intraoperative findings of motor function during awake surgery and obtained limited information with small number of cases. In line with this, our results might be limited to brain tumors growing relatively slow but not to strokes that develop acute onset (Desmurget et al., 2007). As a limitation of DES, we cannot obtain direct evidence with intraoperative mapping outside of the mapping area. However, we believe that our results would be useful for understanding the recovery process in neurological disorder that infiltrates beyond the motor area.

Second, there are some limitations in the fMRI study, such as low time resolution and artifact, which may reduce the accuracy of the result. To compensate for these limitations, multiple-task fMRI or confirmatory tests are needed (Deng et al., 2015). In the current study, we confirmed the results of two types plus the unknown type of motor functional shifts by double methods, DES and fMRI. In line with this, the patients with recurrence were included in the current study, since they were essential for our study to understand the characteristics of motor functional shift. In our institution, titan plate is used to fix bone, and it might be the cause of artifact within an area of a few centimeters from each titan plate. However, we employed a relatively wide range of craniotomy. Hence, the region of interest for fMRI did not overlap on peripheries of craniotomy. Additionally, for higher-grade glioma cases, early surgery was the priority; therefore, we could not perform a full study of MRI preoperatively (Cases 5 and 6).

Third, all positive mapping sites were overlapped on MNI space to compare spatial location of each positive mapping. Brains with resection cavity have the tendency to deform, especially those with large resection cavity. Generally, we exclude patients who could not successfully normalize by any means from the patient group of the study. In the current study, we could normalize postoperative MRI in all patients successfully, and we could identify exact spatial location of positive mapping sites via the procedure mentioned in the *Materials and methods* section.

Fourth, in this study, the functional shift was determined when the motor area moved outside of the original primary motor area. Although it remains unclear whether motor functioning can be found outside of the primary motor area in the normal brain, in our patient group, patients whose tumor cells did not infiltrate into the motor area or adjacent to the motor area did not experience motor functional shift. Finally, the fact that functional connectivity can change in accordance with functional recovery has been demonstrated by various studies (Vassal et al., 2017; Bramati et al., 2019). Although the actual mechanism for the change of structural connectivity corresponding to the change of functional connectivity remains unclear, some reorganization models have been proposed (Duffau, 2009).

## Conclusion

Motor function can be reorganized in two ways, “Move to the ipsilateral different gyrus” and “Move within the ipsilateral precentral gyrus”, and it occurs with high probability under the following two conditions: repeated surgery and when tumor cells infiltrate into the primary motor area. Though our findings of this study might be limited to gliomas or slow progression brain disease, they are expected to have an impact on the field of neuroscience as well as clinicians such as neurology, neurosurgery, and rehabilitation.

## Data Availability Statement

The raw data supporting the conclusions of this article will be made available by the authors, without undue reservation, to any qualified researcher.

## Ethics Statement

The studies involving human participants were reviewed and approved by the Medical Ethics Committee of Kanazawa University. The patients/participants provided their written informed consent to participate in this study.

## Author Contributions

MN and RN conceived and designed the study, and drafted the manuscript. RN and MK acquired, analyzed, and interpreted the data. MN supervised the study. All authors revised and reviewed final version of the manuscript, and approved it for submission.

## Conflict of Interest

The authors declare that the research was conducted in the absence of any commercial or financial relationships that could be construed as a potential conflict of interest.

## Footnotes

1 http://www.fil.ion.ucl.ac.uk/spm/software/spm12/

2 http://www.mathworks.com/products/matlab/

3 http://www.mccauslandcenter.sc.edu/mricro/mricron/

4 https://www.mccauslandcenter.sc.edu/mricrogl/

5 https://www.gehealthcare.com/products/advanced-visualization/all-applications/brainwave

## Supplementary Material

FIGURE S1 1st step **(a)** Each positive mapping sites were plotted on the 3D-brain images which is generated via MR images using iPlan Stereotaxy 3.0 software (BrainLab). The exact locations of the positive mapping sites were determined in accordance with their spatial relationships to various anatomical landmarks, such as gyri, sulci, resection cavity at the previous surgery, and so on. The plotted positive mapping sites on 3D-brain can be also automatically plotted on original 2D-MR images. 2nd step **(b)** Each positive mapping sites were plotted on the normalized T1-images. 3rd step: The spatial location of the positive motor mapping sites were overlaid on the 3D MNI brain using the MRIcroGL software, and then they were re-checked the anatomical location comparing with operative record. A, anterior; P, posterior; M medial. Upper column (A): Black triangles, central sulcus; blue triangles, superior frontal sulcus; star, resection cavity in the first operation; yellow circled region, positive motor responses elicited by the DES. Lower column (B): Black triangles, central sulcus; blue triangle, inferior frontal sulcus; white triangles, sylvian fissure; yellow circled region, positive motor responses elicited by the DES.

FIGURE S2 These images present additional information for Case 7 and 8 in the previous surgery. The left column shows the preoperative MRI, and the middle column shows the preoperative fMRI. The right column presents the final view of the previous surgery. During awake surgery, motor sites were positively mapped in the precentral gyrus in both cases (yellow circled region). Red triangles: central sulcus; broken line in rightward column: central sulcus; NMR: negative motor response; A: anterior; P: posterior; M: medial side.

FIGURE S3 Functional shift depending on surgical history and lesion. We investigated whether the motor area was moving or being stable based on **(A)** the surgical history and **(B)** tumor location. The results revealed that reorganization tended to occur with high probability after the second or subsequent surgeries. When glioma cells expanded into Brodmann’s area (BA) 4, all patients experienced motor functional shift. In contrast, patients whose tumor locations were not in BA 4 or BA 6 did not experience functional shift.

VIDEO S1 Motor area moved within the ipsilateral precentral from original position in Case 6.

VIDEO S2 Negative motor response during electrical stimulation in Case 8.
