# Supplementary figures and images for: Motor Functional Reorganization Is Triggered by Tumor Infiltration Into the Primary Motor Area and Repeated Surgery
Source: Front Hum Neurosci. 2020 Aug 14;14:327. doi: 10.3389/fnhum.2020.00327 (PMC7457049; doi:10.3389/fnhum.2020.00327)

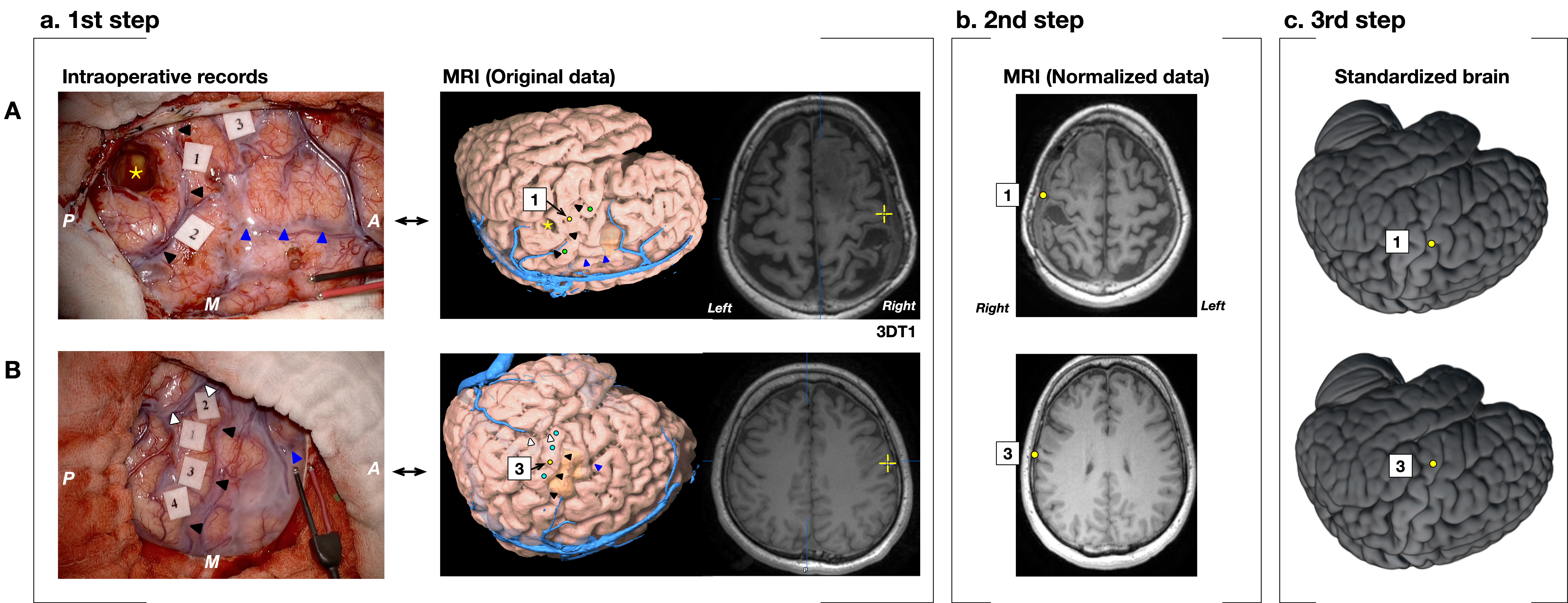

Supplement: FIGURE S1 — 1st step (a) Each positive mapping sites were plotted on the 3D-brain images which is generated via MR images using iPlan Stereotaxy 3.0 software (BrainLab). The exact locations of the positive mapping sites were determined in accordance with their spatial relationships to various anatomical landmarks, such as gyri, sulci, resection cavity at the previous surgery, and so on. The plotted positive mapping sites on 3D-brain can be also automatically plotted on original 2D-MR images. 2nd step (b) Each positive mapping sites were plotted on the normalized T1-images. 3rd step: The spatial location of the positive motor mapping sites were overlaid on the 3D MNI brain using the MRIcroGL software, and then they were re-checked the anatomical location comparing with operative record. A, anterior; P, posterior; M medial. Upper column (A): Black triangles, central sulcus; blue triangles, superior frontal sulcus; star, resection cavity in the first operation; yellow circled region, positive motor responses elicited by the DES. Lower column (B): Black triangles, central sulcus; blue triangle, inferior frontal sulcus; white triangles, sylvian fissure; yellow circled region, positive motor responses elicited by the DES. [file Image_1.TIFF]

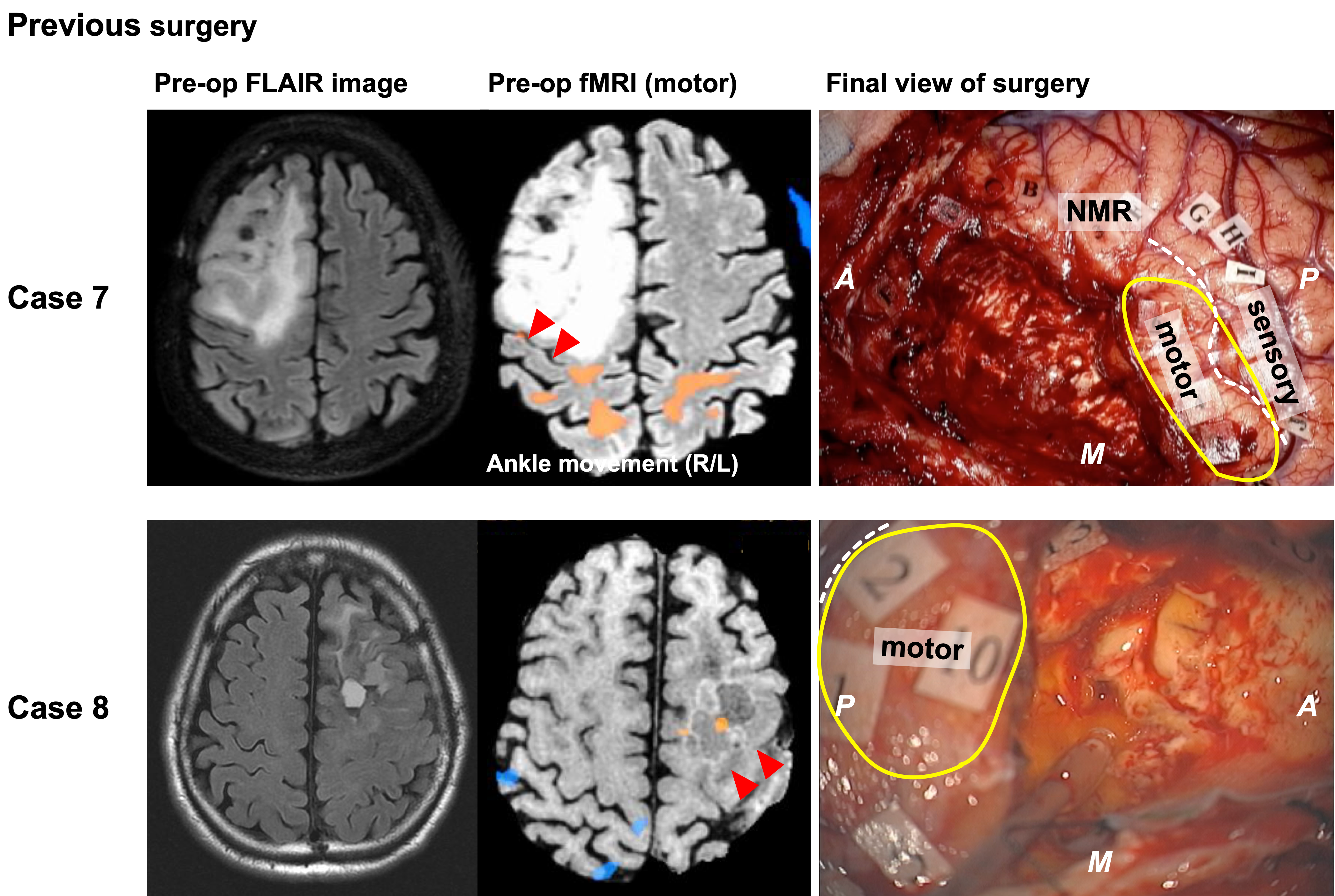

Supplement: FIGURE S2 — These images present additional information for Case 7 and 8 in the previous surgery. The left column shows the preoperative MRI, and the middle column shows the preoperative fMRI. The right column presents the final view of the previous surgery. During awake surgery, motor sites were positively mapped in the precentral gyrus in both cases (yellow circled region). Red triangles: central sulcus; broken line in rightward column: central sulcus; NMR: negative motor response; A: anterior; P: posterior; M: medial side. [file Image_2.TIFF]

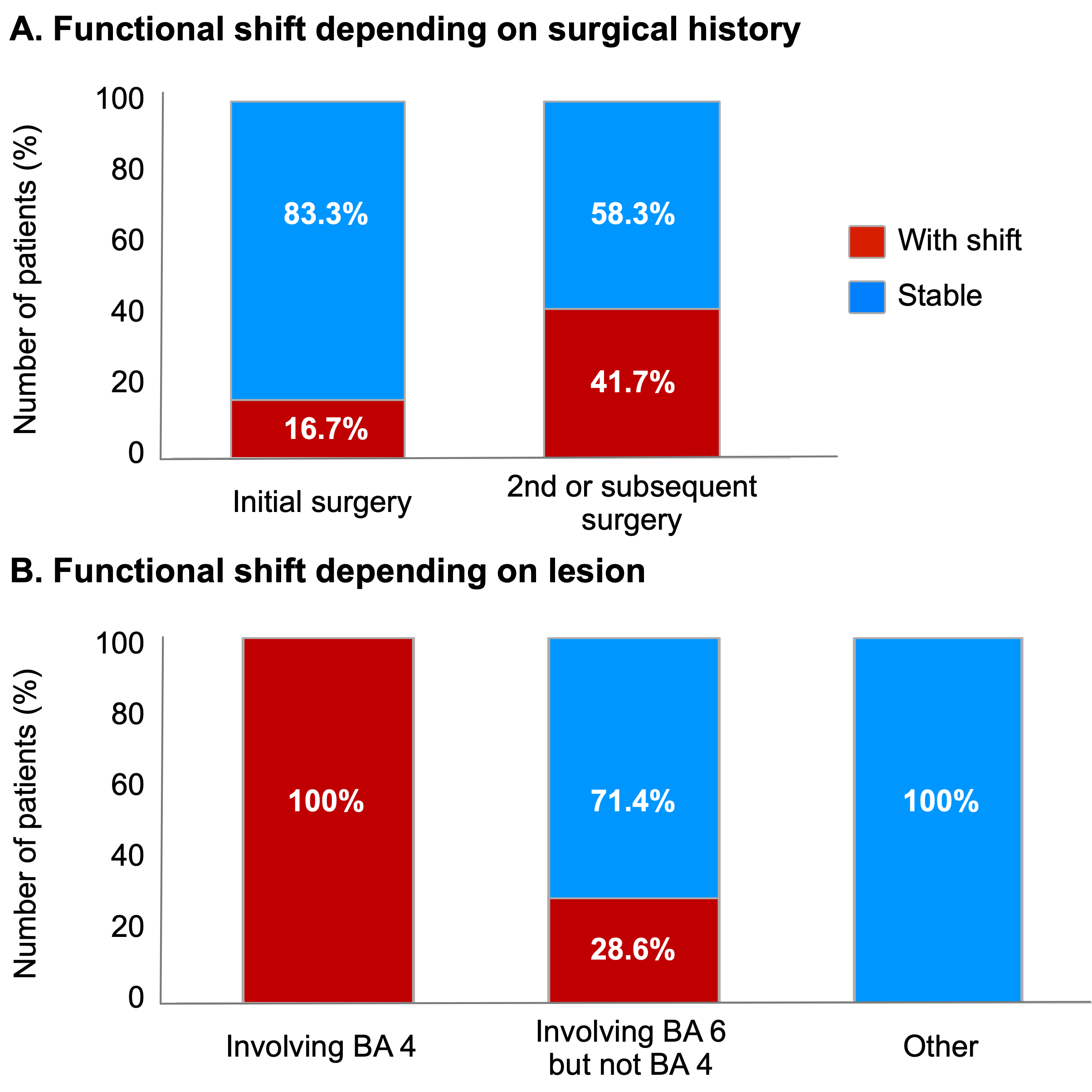

Supplement: FIGURE S3 — Functional shift depending on surgical history and lesion. We investigated whether the motor area was moving or being stable based on (A) the surgical history and (B) tumor location. The results revealed that reorganization tended to occur with high probability after the second or subsequent surgeries. When glioma cells expanded into Brodmann’s area (BA) 4, all patients experienced motor functional shift. In contrast, patients whose tumor locations were not in BA 4 or BA 6 did not experience functional shift. [file Image_3.TIFF]
