# Supplementary material for: Motor Functional Reorganization Is Triggered by Tumor Infiltration Into the Primary Motor Area and Repeated Surgery
Source: Front Hum Neurosci. 2020 Aug 14;14:327. doi: 10.3389/fnhum.2020.00327 (PMC7457049; doi:10.3389/fnhum.2020.00327)
Supplement: Supplementary file 6 [file Table_1.docx]

**Supplementary table 1**. Postoperative course of motor function

| **Case** | **Degree of paresis (BRS score)** | | |
| --- | --- | --- | --- |
|  | **Pre-op** | **Post-op 1 week** | **Post-op 3 months** |
| 1 | Normal | Normal | Normal |
| 2 | Normal | Slightly (V) | Slightly (VI) |
| 3 | Normal | Normal | Normal |
| 4 | Normal | Slightly (V) | Normal |
| 5 | Normal | Moderate (IV) | Slightly (V) |
| 6 | Normal | Slightly (VI) | Normal |
| 7 | Normal | Slightly (VI) | Normal |
| 8 | Normal | Slightly (V) | Slightly (VI) |

BRS, Brunnstrom recovery stage

**Supplementary table 2**. MNI coordinates of positive mapping sites for motor symptom

| **Points’ No.** | **Laterality** | **Response of body part** | **MNI coordinates** | | |
| --- | --- | --- | --- | --- | --- |
|  |  |  | **X** | **Y** | **Z** |
| 1 | R | finger | 48 | -8 | 59 |
| 2 | R | wrist | 42 | -13 | 68 |
| 3 | R | finger | 48 | -9 | 57 |
| 4 | R | elbow | 35 | -17 | 69 |
| 5 | R | elbow | 38 | -19 | 69 |
| 6 | R | elbow | 46 | -27 | 67 |
| 7 | R | elbow | 46 | -27 | 64 |
| 8 | R | elbow | 48 | -32 | 64 |
| 9 | R | elbow | 48 | -24 | 63 |
| 10 | R | elbow | 51 | -24 | 60 |
| 11 | R | elbow | 48 | -14 | 60 |
| 12 | R | elbow | 50 | -11 | 57 |
| 13 | R | elbow | 48 | -9 | 56 |
| 14 | R | elbow | 49 | -8 | 56 |
| 15 | R | elbow | 49 | -15 | 61 |
| 16 | R | elbow | 38 | -18 | 69 |
| 17 | R | elbow | 44 | -17 | 68 |
| 18 | R | wrist | 48 | -8 | 58 |
| 19 | R | elbow | 53 | -17 | 56 |
| 20 | R | face | 58 | -4 | 49 |
| 21 | R | finger | 51 | -5 | 56 |
| 22 | R | wrist | 50 | -1 | 56 |
| 23 | R | elbow | 35 | -22 | 71 |
| 24 | R | elbow | 22 | 2 | 43 |
| 25 | R | wrist | 39 | -24 | 69 |
| 26 | L | finger | -43 | -20 | 65 |
| 27 | L | face | -64 | -10 | 27 |
| 28 | R | face | 61 | 3 | 36 |
| 29 | R | face | 60 | -2 | 42 |
| 30 | R | face | 66 | -2 | 23 |
| 31 | L | wrist | -49 | -12 | 56 |
| 32 | L | elbow | -42 | -18 | 64 |
| 33 | L | wrist | -41 | -23 | 68 |
| 34 | L | wrist | -36 | -23 | 71 |
| 35 | L | wrist | -43 | -16 | 63 |
| 36 | L | elbow | -55 | -7 | 49 |
| 37 | L | elbow | -52 | -7 | 53 |
| 38 | L | elbow | -47 | -25 | 64 |
| 39 | L | elbow | -42 | -10 | 64 |
| 40 | L | elbow | -42 | -30 | 69 |
| 41 | L | elbow | -52 | -9 | 52 |
| 42 | L | elbow | -56 | -7 | 49 |
| 43 | L | elbow | -40 | -14 | 67 |
| 44 | L | elbow | -37 | -19 | 69 |
| 45 | L | elbow | -45 | -17 | 66 |
| 46 | L | foot | -45 | -24 | 66 |
| 47 | L | foot | -12 | -24 | 41 |
| 48 | L | foot | -12 | -27 | 47 |
| 49 | L | foot | -4 | -26 | 55 |
| 50 | L | wrist | -52 | -7 | 51 |
| 51 | L | face | -51 | -10 | 55 |
| 52 | L | wrist | -54 | -3 | 48 |
| 53 | R | face | 31 | 7 | 33 |
| 54 | R | finger | 20 | 2 | 53 |
| 55 | R | finger | 19 | 2 | 33 |
| 56 | R | finger | 43 | -20 | 68 |
| 57 | L | elbow | -29 | -27 | 42 |
| 58 | L | foot | -22 | -36 | 38 |
| 59 | R | elbow | 40 | -22 | 68 |
| 60 | L | face | -57 | -2 | 44 |
| 61 | R | elbow | 36 | -22 | 72 |
| 62 | R | wrist | 33 | -13 | 71 |
| 63 | R | wrist | 41 | -20 | 70 |
| 64 | R | wrist | 33 | -12 | 71 |
| 65 | R | elbow | 16 | -4 | 46 |
| 66 | R | elbow | 16 | -7 | 43 |
| 67 | R | elbow | 46 | -18 | 64 |
| 68 | R | face | 59 | -4 | 41 |
| 69 | R | face | 60 | 0 | 39 |
| 70 | R | finger | 31 | -14 | 71 |
| 71 | R | finger | 35 | -11 | 69 |
| 72 | R | finger | 43 | -20 | 66 |
| 73 | R | finger | 44 | -14 | 63 |

R, Right; L, left
